# A core genome multi-locus sequence typing scheme for Streptococcus uberis: an evolution in typing a genetically diverse pathogen

**DOI:** 10.1099/mgen.0.001225

**Published:** 2024-03-21

**Authors:** Daniel Whiley, Keith Jolley, Adam Blanchard, Tracey Coffey, James Leigh

**Affiliations:** 1 School of Veterinary Medicine and Science, University of Nottingham, Nottingham, UK; 2 Department of Biology, University of Oxford, Oxford, UK

**Keywords:** core genome, multi-locus sequence typing, phylogeny, sequencing, *Streptococcus uberis*

## Abstract

*Streptococcus uberis* is a globally endemic and poorly controlled cause of bovine mastitis impacting the sustainability of the modern dairy industry. A core genome was derived from 579 newly sequenced *S. uberis* isolates, along with 305 publicly available genome sequences of *S. uberis* isolated from 11 countries around the world and used to develop a core genome multi-locus sequence typing (cgMLST) scheme. The *S. uberis* core genome comprised 1475 genes, and these were used to identify 1447 curated loci that were indexed into the cgMLST scheme. This was able to type 1012 of 1037 (>97  %) isolates used and differentiated the associated sequences into 932 discrete core genome sequence types (cgSTs). Analysis of the phylogenetic relationships of cgSTs revealed no clear clustering of isolates based on metadata such as disease status or year of isolation. Geographical clustering of cgSTs was limited to identification of a UK-centric clade, but cgSTs from UK isolates were also dispersed with those originating from other geographical regions across the entire phylogenetic topology. The cgMLST scheme offers a new tool for the detailed analysis of this globally important pathogen of dairy cattle. Initial analysis has re-emphasized and exemplified the genetically diverse nature of the global population of this opportunistic pathogen.

**Impact Statement**

The ability to type bacteria isolated from disease accurately is paramount in understanding transmission of infection. In the case of *Streptococcus uberis*, a common cause of intramammary infection leading to mastitis in dairy cattle, accurate and precise typing will aid the formulation of new measures to enhance control of this globally endemic disease. Multi-locus sequence typing (MLST), developed for *S. uberis* in 2006, has been used widely in the epidemiology of *S. uberis*. MLST indexes the sequence variation of seven gene fragments to identify strains and genetic relationships among strains. Bacterial genomes contain a core set of genes encoding intrinsic/required functions (core genome) that define the species and additional genes (accessory genome), which may only be present in a limited number of strains within the species. We have exploited the ability to rapidly sequence bacterial genomes to define the core genes of *S. uberis* to establish a new typing scheme (core genome MLST) offering the benefits of standard MLST, but with greater resolution and precision by indexing sequence variation in 1447 core genes of *S. uberis*. The curated database can be interrogated, and new data uploaded by other investigators, providing a new globally applicable tool to investigate this pathogen.

## Data Summary

The authors confirm all supporting data, code and protocols have been provided within the article or through supplementary data files.

## Introduction

*Streptococcus uberis* is an important agent of bovine mastitis worldwide and the most common cause of the disease in the UK [1]. Typing schemes are important in the surveillance of bacterial pathogens and in elucidating transmission routes and bacterial reservoirs/sources of infection. This is particularly the case for *S. uberis* as transmission to the mammary gland from environmental sources is the major route of infection, although cow to cow transmission can also occur [2]. Detailed understanding of the relative importance of environmental reservoirs might aid in the management of dairy cows and the control of infection by this organism. Similarly, accurate confirmation of transmission of an infectious clone may indicate issues with milking time hygiene and/or biosecurity.

There have been multiple examples of typing tools developed for *S. uberis*, such as RAPD [3], repetitive extragenic palindromic PCR [4], genomic DNA RFLP methodologies [5,6], multiple loci variable number of tandem repeat typing [7], lytic phage typing [8] and serotyping [9]. These phenotypic, molecular and non-molecular approaches are labour intensive, costly and unable to provide an objective and reliable methodology to differentiate closely related strains [10]. Furthermore, in many of the techniques the outputs are not portable and do not facilitate comparison of data between laboratories.

Sequence-based typing techniques have also been applied to *S. uberis* [11] and the multi-locus sequence typing (MLST) [12] scheme using seven housekeeping loci, developed in 2006, is hosted online on the PubMLST server (https://pubmlst.org). MLST is fully portable and additive and has been widely used to investigate phylogenetic relationships of *S. uberis* [13,17]. The existing MLST scheme indexes genetic relationships but contains many singletons [sequence types (STs) that do not show clonal relationships with others within the scheme]. In addition, two of the seven loci used in this scheme have been shown to be absent from some isolates, although there is some indication that one clonal complex based on ST86 may be associated with lower virulence [18].

Core genome MLST (cgMLST) schemes, comprising genes found in the majority of sequenced isolates [19], have become available for other bacterial species [20]. These schemes have successfully typed many different bacterial pathogens [21,24]. Due to the increased amount of data used for typing, greater heterogeneity of each profile is permitted compared to MLST. Therefore, it is anticipated that cgMLST will enable comparison of even distantly related variants of *S. uberis*, currently identified as singletons in the existing scheme. In addition, the increased data will also offer higher resolution than the existing scheme. Core genome analysis by MLST retains all the key aspects of portability and data accumulation. In an era where whole genome sequencing of bacterial isolates has become routine, such schemes have become increasingly important and cost-effective tools for investigation of bacterial pathogens.

In this study, we publish an additional 579 genomes of *S. uberis*, more than doubling the number publicly available in the PubMLST *S. uberis* database [12,25]. Using the entirety of these genome data we developed a cgMLST scheme and compared this against the existing MLST scheme.

## Methods

### Culture collection and isolation of new *S. uberis* isolates

A total of 579 isolates of *S. uberis* obtained from collections accumulated by the authors were used in this study. Of these isolates, 29 were of environmental origin while the remaining 550 isolates were obtained from bovine milk. In this culture collection, 567 isolates were of UK origin while the remaining 12 isolates were from Denmark. Isolates were collected between the years 1970 and 2022 (median: 2002).

For genomic sequencing, the *S. uberis* isolates were cultured for purity on Blood Agar Base with 5 % sheep blood including 0.1 % aesculin and incubated at 37 °C for ~18 h in aerobic conditions. A single colony isolate was picked from each culture plate into Todd-Hewitt broth and cultured for a further 18 h at 37 °C. Approximately 2 ml of an OD_600_ 1.0 culture was taken forward for DNA extraction while the remaining culture was mixed with sterile glycerol (25  %, v/v, final concentration) and stored at −20 °C.

### *S. uberis* DNA extraction and whole genome sequencing

DNA was extracted following the methods published by Hill and Leigh [26]. Genomic DNA for each isolate used within this study was sent for whole genome sequencing at the Leeds Institute of Molecular Medicine, University of Leeds. Paired-end NEBNext Ultra DNA libraries were generated and sequenced on the Illumina MiSeq platform and set to sequence in 250 bp paired-end mode using V2 chemistry.

One strain, SUD69, was additionally sequenced on the MinION (Oxford Nanopore Technologies) platform following the manufacturer’s instructions using the R9.4.1 flow cell and following the Native Barcoding library preparation protocol (version NBE_9065_v109+_revAD_14Aug2019).

### Sequencing read curation, whole genome assembly and annotation

Illumina sequencing reads were trimmed in paired-end mode by FastP [27] (version 0.20.2) to remove sequencing adapters, trim ends to Phred 30 and remove reads smaller than 20 bp in length. Reads were passed through FastQC (https://github.com/s-andrews/FastQC) (version 0.11.9) to ensure correct trimming had occurred. Curated reads were passed through Kraken2 [28] (version 2.1.2) using the standard bacterial database (built on 27 January 2021) to ensure >90  % of reads were associated with *S. uberis*. Samples with genomic reads that were suspected to not be from *S. uberis* were removed from the dataset. Samples passing these quality control checks were *de novo* assembled through Shovill (https://github.com/tseemann/shovill) (version 1.1.0) with an estimated genome size of 1.93 Mb and in paired-end mode. Isolate SUD69 was assembled following a hybrid long- and short-read methodology as previously described [29]. Genomic assemblies were assessed for quality through QUAST [30] (version 5.0.2) for confirmation of expected assembly indicators (genome size <2.4 Mb, number of contigs <450, GC content 36–7  %). Genomes were annotated through Prokka [31] (version 1.14.6) guided by the *S. uberis* 0140J reference (NC_012004.1) using default parameters in compliant mode.

### Determining the pan-genome of all *S. uberis* isolates sequenced in this study

Pan-genome analysis was performed using Panaroo [32] (version 1.2.7) on all newly sequenced isolates in this study (*n*=579) along with the virulent and avirulent reference strains 0140J (NC_012004.1) and EF20 (NZ_JANW01000001) respectively. Panaroo was run in moderate mode with a core genome threshold set to 99 %.

### Depositing strains to the PubMLST *S. uberis* BIGSdb database and the NCBI Sequencing Read Archive (SRA)

The genome assemblies for the 579 genomes generated in this study were uploaded to the PubMLST *S. uberis* database (https://pubmlst.org/organisms/streptococcus-uberis) and unique BIGSdb strain IDs were assigned (Table S1, available in the online version of this article). Additionally, sequencing reads were uploaded to the SRA on the NCBI server under BioProject ID PRJNA1024663.

### MLST and publicly available isolates used in this study

MLST (seven loci) profiles and STs were assigned to each new sequence generated in this study (*n*=579). Publicly available *S. uberis* isolates were downloaded from the PubMLST database in two batches. The first batch of isolates (*n*=305) containing sequencing data was downloaded on 15 February 2023 and was used to determine the *S. uberis* core genome (see below) including the 579 isolates published in this study. Alongside the reference genomes 0140J (NC_012004.1) and EF20 (NZ_JANW01000001) [33], these isolates were used to determine the core genome of *S. uberis* (isolates denoted in Table S1). An additional 164 isolates were downloaded from PubMLST, which represented newly uploaded *S. uberis* genomes captured between 15 February 2023 and 11 August 2023.

All publicly available genomes with whole genome assembly data available (from 456 isolates) were annotated as previously described through PROKKA [31] to ensure coding sequencing annotation nomenclature was consistent between isolates.

### Identification of the *S. uberis* core genome and development of the cgMLST scheme

Core genes were identified using the BIGSdb Genome Comparator plugin [25] (version 2.7.7) by comparing the whole genome MLST (wgMLST) profiles of the newly sequenced *S. uberis* isolates in the study (*n*=579) as well as 307 isolates with sequencing data downloaded from the PubMLST *S. uberis* BIGSdb database on 15 February 2023 (including the reference strains 0140J and EF20). The blastn parameters were set to a minimum nucleotide identity of 70 %, a minimum alignment of 90 % and a word size of 20 bp. The initial core gene set was determined as genes present in 99 % of isolates.

The set of core genes were further analysed for problems that could impact the final scheme. The start positions of some loci were modified slightly where it was clear that the position indicated in the original reference sequence was not conserved across the population dataset and an alternative conserved start codon was observed nearby. In other cases, loci with internal stop codons in some isolates, where a stop codon was not consistently found or where a gene was found to have a paralogue that could result in multiple allele designations were also removed. A total of 28 loci were removed from the core genome set and not included in the final scheme for these reasons (Table S2).

### Gene functional analysis

EggNOG-mapper [34,35] (version 2.1.9) for genome-wide functional annotation was used against the protein sequences from the *S. uberis* 0140J reference strain (NC_012004.1). Clusters of orthologous genes (COG) categories were defined for each gene in the genome, and where a COG category could not be assigned, it was designated as S (function unknown).

### Comparison of standard and core genome MLST

Minimum spanning trees based on standard and cgMLST profiles generated by BIGSdb were visualized in GrapeTree [36] version 1.5.0.

## Results

### Curation of isolates used in this study

Some isolates were removed from the dataset (Table S1). The sequences of six isolates were shown to contain data identifying with multiple streptococcal species using the ribosomal MSLT (rMLST) scheme [37], and seven isolates were shown to have unusually high genome lengths (2.4–2.8 Mb). The final list of curated isolates (*n*=1037) originated from 11 different countries (Table S1).

**Fig. 1.**
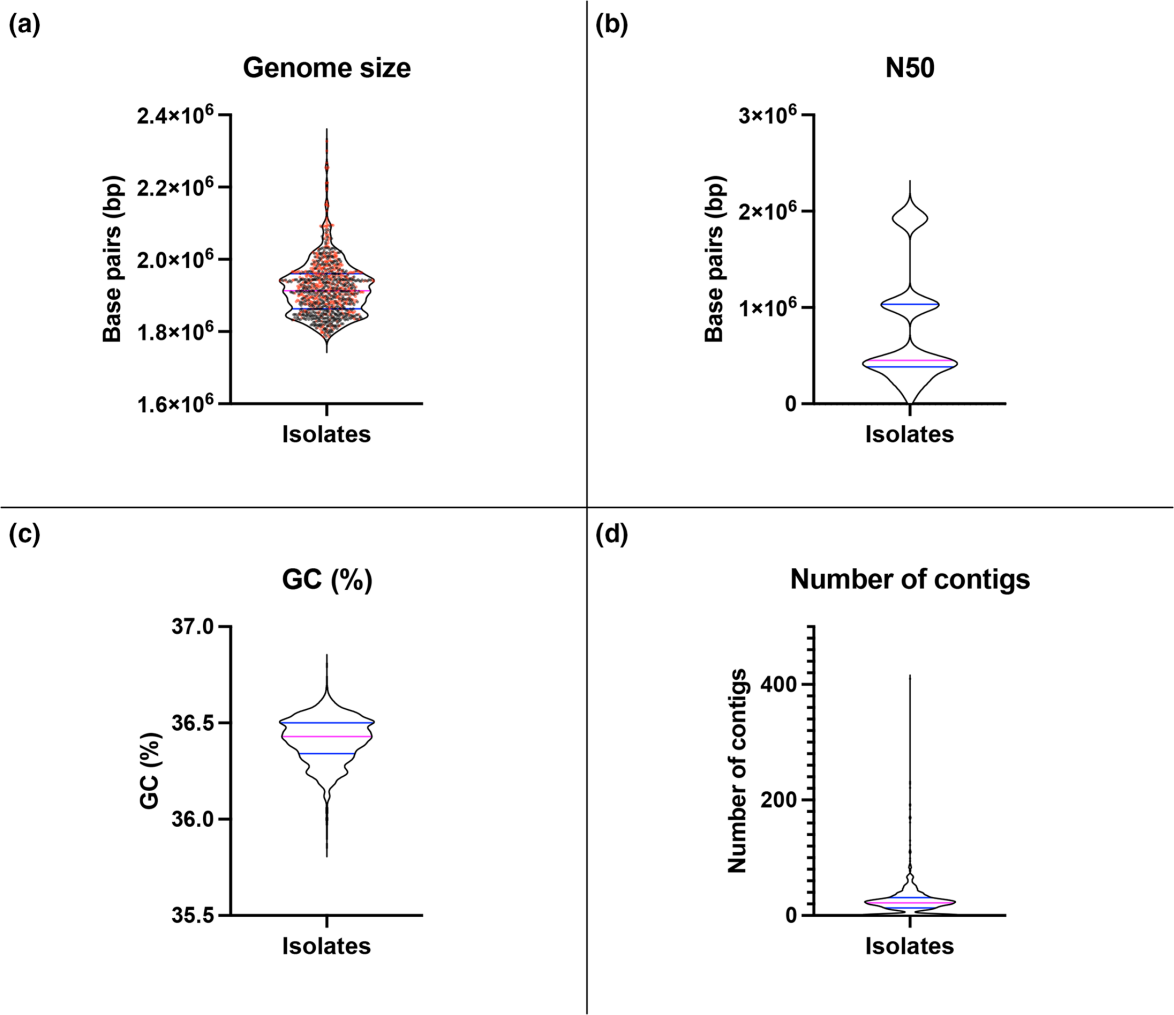
Assembly statistics for all 1037 *S*. *uberis* isolates used within this study. Violin plots of key assembly statistics of all isolates used within this study (579 newly sequenced isolates and 458 publicly available genome sequences). (a) The genome sizes of all isolates. (b) N50 of all isolates. (c) Percentage of GC bases in the genomes. (d) Number of contigs that make up the genome assemblies of each isolate. The median (magenta line) and first and third quartiles (blue lines) are displayed in each plot. Violin plots were created in Prism version 10.0.2 and the genome statistics were determined through QUAST as described in the Methods.

The newly sequenced isolates (*n*=579) had an average and median genome length of 1.9 Mb and were assembled into between one (strain SUD69) and 410 (strain SUD542) contigs (median 26), and on average the N50 of assembled isolate genomes was 0.53 Mb (median 0.43 Mb) in length and had an average GC content of 36.45 % (median 37.47 %). These assembly statistics were in line with the publicly available genome sequence data from other isolates used in this study (Fig. 1).

### Analysis of pan- and core genome sequences of *S. uberis*

The core genome defined by the Genome Comparator plugin on the BIGSdb database consisted of 1475 genes (*n*=886 isolates). The core genome of all isolates newly sequenced in this study, defined by Panaroo, consisted of 1531 genes (*n*=581 isolates). A total of 6690 genes were identified in the Panaroo analysis, with the pan-genome broken down into 4694 cloud genes (found in 0–15  % of isolates), 401 shell genes (found in 15–95 % of isolates) and 64 soft core genes (found in 95–99 % of isolates).

### Development of the cgMLST scheme

**Fig. 2.**
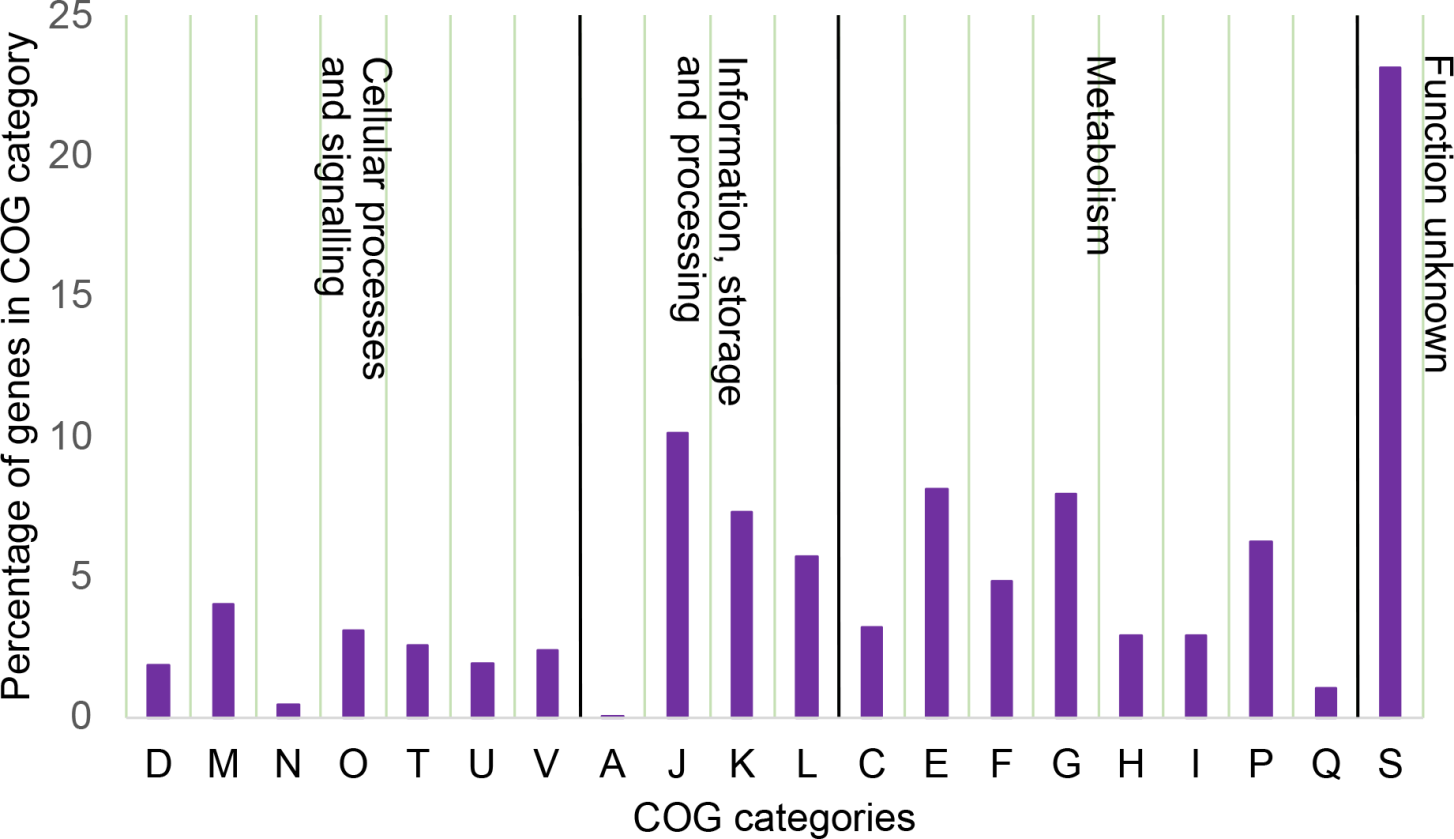
Clusters of orthologous genes (COG) categories of the cgMLST scheme genes. The genes that make up the core genome were binned into COG categories and depicted as a bar chart. The COG categories were further grouped by their general definitions, Cellular processes and signalling, Information, storage and processing, Metabolism, and function unknown, and are separated by black lines in the figure. The COG categories are as follows: A, RNA processing and modification; C, energy production and conversion; D, cell cycle control, cell division, chromosome partitioning; E, amino acid transport and metabolism; F, nucleotide transport and metabolism; G, carbohydrate transport and metabolism; H, coenzyme transport and metabolism; I, lipid transport and metabolism; J, translation, ribosomal structure and biogenesis; K, transcription; L, replication, recombination and repair; M, cell wall/membrane/envelope biogenesis; N, cell motility; O, post-translational modification, protein turnover and chaperones; P, inorganic ion transport and metabolism; Q, secondary metabolite biosynthesis, transport and catabolism; S, function unknown; T, signal transduction mechanisms; U, intracellular trafficking, secretion and vesicular transport; and V, defence mechanisms.

The curated cgMLST scheme consisted of 1447 loci (excluded loci are detailed in Table S2). A core genome ST (cgST) was assigned to sequences in which 25 or fewer loci were missing – this threshold was chosen to provide a robust scheme that allowed the majority of well-assembled genomes to be assigned while minimizing the artefacts that missing loci can have on clustering. A total of 1012 (97.6  %) of the 1037 isolates were assigned a cgST. Twenty-five of the 1037 isolates could not be assigned a cgST, as 21 had 26–100 missing loci and four had more than 100 missing loci. The 1012 typed isolates were assigned to 932 cgSTs, indicating that 84 (8.4  %) shared a common clonal origin with others in the database. The loci that constituted the cgMLST scheme grouped into four broad categories: metabolic genes (37.3 %), information storage and processing genes (23.2 %), poorly characterized genes (23.2 %), and cellular processes and signalling genes (16.3 %) (Fig. 2).

To ensure that the cgMLST scheme could be applied to new assemblies without extensive manual curation, a set of 164 isolates downloaded from PubMLST between 15 February 2023 and 11 August 2023 (not included in the core genome development set) were typed with the scheme; 161 (>97.5  %) of these were successfully assigned a cgST (Table S1). This was in line with the percentage of isolates typed during the development of the cgMLST scheme.

### Comparison of MLST and cgMLST analysis

Of the 1037 *S*. *uberis* isolates analysed, there were a total of 458 STs and 931 cgSTs. MLST failed to define STs for nine isolates due to missing loci other than the permitted absence of loci for *yqil* and *recP*. The newly sequenced isolates (*n*=579) contained 215 novel STs and 525 novel cgSTs.

The most prevalent ST was ST6 (*n*=75 isolates) followed by ST5 (*n*=61), ST20 (*n*=47), ST26 (*n*=39) and ST23 (*n*=23), with the remaining STs identified in 18 or fewer isolates. The cgMLST scheme was able to resolve each of the prevalent STs into 61, 53, 44, 25 and 15 cgSTs, respectively. The 10 most prevalent STs accounted for 30.87 % of all isolates with a defined ST (*n*=1028). In contrast, the 10 most prevalent cgSTs comprised only 4.84 % of isolates with a defined cgST (*n*=1012).

### cgMLST analysis

**Fig. 3.**
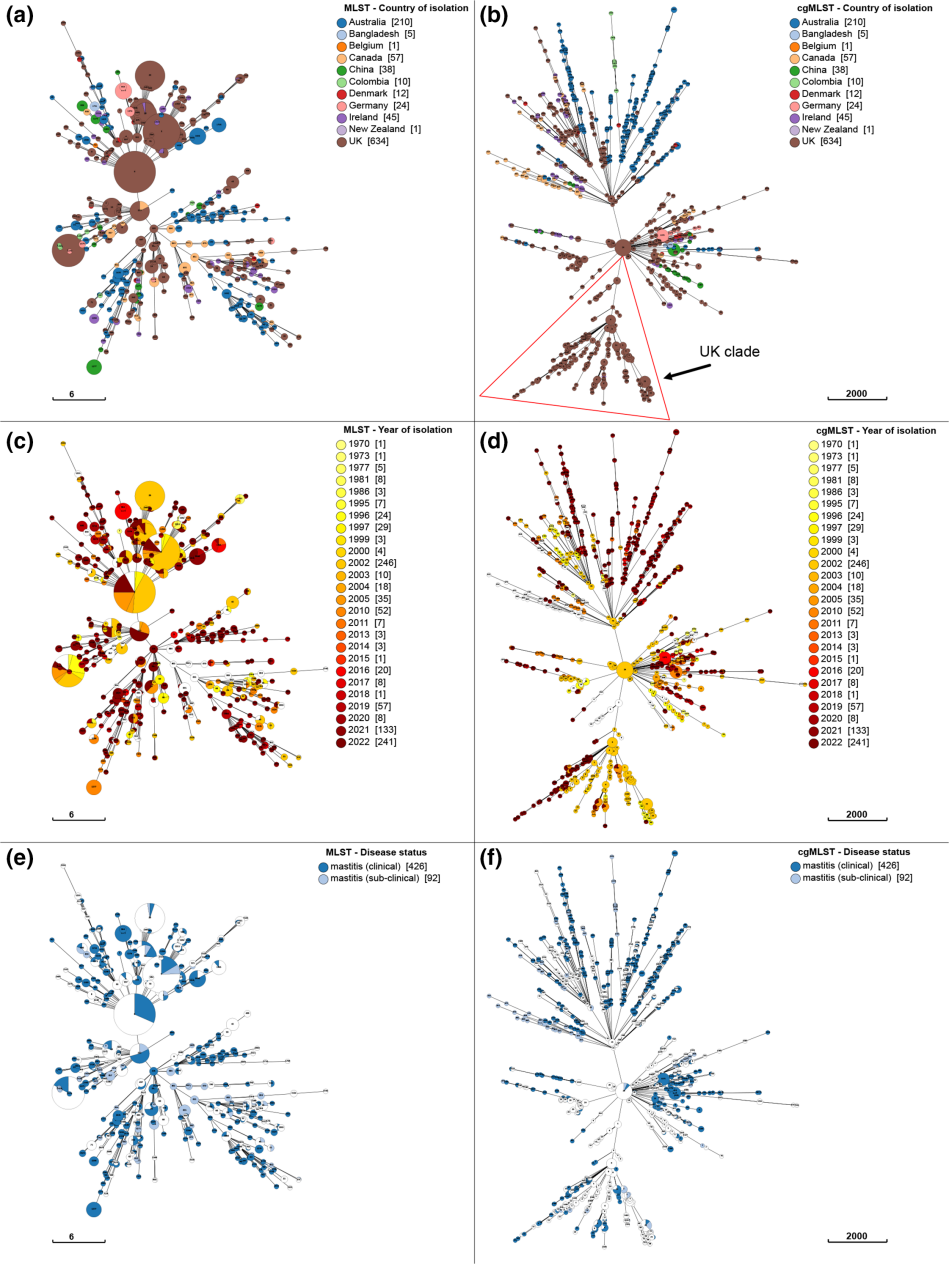
Comparison of the ST profiles and the cgST profiles with associated metadata. MLST and cgMLST cluster comparisons through GrapeTree using all 1037 isolates in this study. The metadata for country of isolation (a, b), year of *S. uberis* isolation (c, d) and disease status (e, f) are depicted. Allele designations and metadata were harvested from PubMLST. White colours designate isolates with missing metadata. For each panel (a–f) the ST designation is denoted within each cluster. These were included to aid interpretation of the results and to allow direct comparisons between the two schemes. The scale bar for each figure refers to the number of alllelic differences.

Although the cgMLST scheme produced higher resolution of typing than MLST (Fig. 3), based on visual inspection no greater or obvious alignment of cgSTs or single gene linkage clusters with any of the associated and routinely collected metadata (disease status, year of isolation) was evident (Fig. 3a–f).

However, cgMLST provided some association regarding country of isolation that was not evident using MLST. Isolates from the UK and Australia (the most prevalent in the database: 634 and 210, respectively) were present throughout the phylogenetic tree (Fig. 3b), and some UK or Australian dominant branches were evident. One example is denoted as a UK clade (Fig. 3b). There are many short branches across the topology that only consist of isolates from a specific country of origin, but these often also shared the same year of isolation suggesting some confounding circumstances. However, the isolates within the UK-centric clade (Fig. 3b) were obtained from a variety of sources in the UK, the exception being one isolate obtained from Ireland [38,39], over a 26 year period (1996–2022).

## Discussion

Whole genome sequencing of bacterial pathogens is becoming more common, with the reduction in costs and new approachable technologies coming to the market. The costs to sequence an entire bacterial genome and the costs to sequence a subset of genes, such as the genes for an MLST scheme, are likely to be similar but whole genome sequencing benefits from an increase in usable data. As a result, it is important that tools are developed that are better able to take advantage of shifts in technology. To this end, we have expanded on the *S. uberis* MLST scheme developed in 2006 by Coffey *et al*. [12] and developed a cgMLST scheme based on 1447 indexed loci.

These loci were determined by identifying the core genes in 886 *S*. *uberis* isolates with whole genome sequencing data available on the PubMLST database (307 existing genome sequences and a further 579 newly sequenced isolates published in this study). To improve the usability of this cgMLST scheme, a core gene was defined as present in 99 % of all genomes, having a minimum nucleotide identity of 70 % and an alignment of at least 90 %. These parameters were chosen to allow for a degree of flexibility in calling the core genome, such as avoiding reference gene bias by permitting nucleotide variability and inclusion of partial genes [39]. We further curated the core genome to exclude paralogous genes and genes with internal stop codons. Other studies have estimated the *S. uberis* core genome to contain between 1421 and 1567 genes [11,13, 15, 33, 40, 41] with disparities probably being due to the methodologies employed, parameters used and the number of isolates included. This was also evident in this study where the subset of newly sequenced isolates (*n*=579) were determined to have a core genome size of 1531 genes when calculated by a alternative bioinformatics tool, Panaroo [32], which employs a different methodology to define the core genome. The core genome used within the cgMLST scheme falls within the range of these previous estimates of the *S. uberis* core genome.

Despite evidence of the missing *yqiL* gene fragment in some previous MLST analyses of *S. uberis* [18], the *yqiL* gene was identified as part of the core genome in this study, suggesting that the absence of thi*s* locus is rare (<1  % of isolates). However, the *recP* locus, which is part of the original MLST scheme, was not deemed part of the core genome in this study, due to it being absent in >1  % of isolates. Further interrogation is required to determine if this locus is definitively absent in some isolates or if its absence is an artefact of sequencing and/or genome assembly methodologies. However, the *recP* locus was removed from the cgMLST scheme due to these observations. As a result of these findings, we have now implemented flexibility into the standard MLST scheme to allow for the absence of either the *yqiL* or *recP* locus where absence is designated as allele 0 (zero).

As was the case when developing the MLST scheme, the cgMLST scheme uses the Sanger Institute sequence of the commonly used strain 0140J [42] (NC_012004.1) as the primary template for gene designations and these sequences are anchored at allele 1 for all genes. In designing this scheme, care was taken to remove paralogous gene pairs and genes with internal stop codons represented in the entire collection of isolates. cgSTs can be generated for any sequence providing there are 25 or fewer missing loci. The preceding steps were taken to ensure the usability and longevity of the *S. uberis* cgMLST scheme. Another important consideration is that as more genomes are sequenced and added to PubMLST, the designation of core genes is likely to become smaller over time if a very strict threshold is used. One such example has already been discussed regarding the *recP* locus that was initially designated as a housekeeping gene when the initial MLST scheme was developed (prior to the first *S. uberis* genome sequence being available). Allowing for 25 missing loci builds flexibility into the cgMLST scheme to call cgSTs while maintaining the scheme’s integrity and improved downstream analysis.

A total of 458 STs were designated by the MLST scheme, and 26 isolates were unable to be typed. In comparison, the cgMLST scheme identified 932 cgSTs, with 25 isolates unable to be typed, providing a higher resolution of isolate sequence typing. The high number of different cgSTs (*n*=932) within a population of 1037 isolates continues to support the view that *S. uberis* is an opportunistic pathogen with an array of diverse genotypes able to infect the bovine mammary gland [11,16, 33]. The absence of cgST clustering with disease status (Fig. 3) further supports this view.

Although there was no clear separation of phylogeny by country of isolation (Fig. 3), certain country-specific relationships were evident and multiple small clusters of cgSTs that originated from specific countries (Fig. 3b) were detected. These were often separated by cgSTs originating from isolates obtained from different countries. The clearest relationship between phylogeny and country of origin was the observation of a UK-dominant clade (Fig. 3b), where all but one isolate originated from the UK, the exception being from Ireland. Clustering of some cgSTs may be artefactual, relating to sampling. For instance, isolates derived from the same dairy herd over a short time period or from an individual study over a limited geographical area may be closely related through progeny and mutation and limited genetic recombination [13,15, 33]. This is supported in the data we present as isolates within small geographically related clusters/branches also share the same year of isolation. The UK-dominant clade identified represented isolates obtained from various sources over a period of 26 years, with such clustering over longer periods more likely to represent the true abundance of certain related genotypes. However, *S. uberis* cgSTs of UK origin (and to a lesser extent those from Australia) were also spread throughout the phylogenetic tree, spanning numerous clades. This pattern may be expected due to the dominance of UK (and Australian) isolates within the typed population. Overall, the current data support the observation of no obvious population structure based on the country of origin (Fig. 3b) and is indicative of a largely global population. Small geographically linked clusters/branches are probably related due to temporal frequency of isolations within the same geographical area, or use of a limited number of dairy herds in a particular investigation. The cgMLST scheme will provide the means to investigate and challenge this current position.

The cgMLST scheme did not support the differentiation of isolates based on reported disease manifestation (clinical and subclinical mastitis) (Fig. 3f). Isolates obtained from subclinical and clinical mastitis were dispersed across the topology of the minimum spanning tree. However, there were some short branches of isolates relating to clinical or subclinical mastitis, but again there is a possibility these are likely to be artefacts of sampling/study design. Disease status is further complicated by the potential for subclinical cases to progress to a clinical manifestation [43,44] and there are no hard definitions of what constitutes a clinical episode thus further blurring definitive classification of these metadata. Although different strains have been identified as more or less able to infect the mammary gland and cause mastitis [33,45], the absence of a definitive virulent lineage within the cgMLST further supports the concept that intramammary infection in the field is largely opportunistic and not due to circulation (through host and environmental reservoirs) of particularly virulent clones.

## Conclusions

While the existing MLST scheme successfully types *S. uberis* isolates, it is not possible to discriminate closely related isolates within an ST. We have developed a cgMLST scheme based on 1447 loci/genes. This cgMLST scheme provides a high-resolution analysis of *S. uberis* isolates and revealed the high genetic diversity within the species, but also implied a level of genetic relatedness between isolates obtained from different countries. This provides an evolution of the existing and well-used tool for the genetic analysis of *S. uberis*, an important global pathogen of dairy cattle.

## supplementary material

10.1099/mgen.0.001225 Uncited Table S1.
